# Associations between (sub) clinical stress- and anxiety symptoms in mentally healthy individuals and in major depression: a cross-sectional clinical study

**DOI:** 10.1186/s12888-020-02836-1

**Published:** 2020-09-01

**Authors:** Georgia Konstantopoulou, Theodoros Iliou, Katerina Karaivazoglou, Gregoris Iconomou, Konstantinos Assimakopoulos, Panagiotis Alexopoulos

**Affiliations:** 1 Special Office for Health Consulting Services and Faculty of Education and Social Work, School of Humanities and Social Sciences, University of Patras, University of Patras Rion Campus, Rion Patras, 26504 Patras, Greece; 2 Medical Informatics Laboratory, Faculty of Medicine, School of Health Sciences, Democritus University of Thrace, Alexandroupolis Campus, 6th km Alexadroupolis – Makris (Dragana), Alexandroupolis, Greece; 3 Child and Adolescent Psychiatrist, Day Centre for Children with Autism Spectrum and other Developmental Disorders, K. Tzavela 12, 30200 Messolonghi, Greece; 4 Department of Psychiatry, Patras University General Hospital, Faculty of Medicine, School of Health Sciences, University of Patras, PGNP 26504, Rion, Patras, Greece; 5 Department of Psychiatry and Psychotherapy, Klinikum rechts der Isar, Faculty of Medicine, Technical University of Munich, Ismaninger Str. 22, 81675 Munich, Germany

**Keywords:** Subclinical stress symptom questionnaire-25, Beck anxiety inventory, Positive association

## Abstract

**Background:**

Responses to stressful circumstances have psychological and physiological dimensions, and are related to anxiety symptoms and mental disorders such as depression. Nonetheless, the relationship between subclinical stress and anxiety symptoms is still elusive.

**Methods:**

To explore possible associations between stress and anxiety symptoms, patients with major depression (*N* = 77) and mentally healthy individuals of different age clusters and occupations (*N* = 412) were enrolled into the study. Stress was assessed with the new subclinical stress symptom questionnaire (SSQ-25). Anxiety was studied with the Beck Anxiety Inventory (BAI), mainly focusing on clinical anxiety, whilst anxiety as a personality trait was assessed with the trait aspect of the State Τrait Αnxiety Ιnventory Y (STAI Y). Statistical analyses included ANOVA, Scheffe test, linear regression models and a two-step cluster analysis using Log-Likelihood Distance measure and fixed number of two clusters.

**Results:**

Age, stress symptoms and BAI scores differed significantly between among groups (*P* < 0.001), whilst STAI Y scores did not. Stress levels were found to be related to clinical anxiety (*P* < 0.001), while neither group identity nor age exerted any influence on anxiety levels (*P* > 0.05). The two Step Cluster analysis classified 76 out of 77 participants with milder stress (subclinical) symptoms into the cluster with moderate anxiety, as indicated by BAI scores, and all individuals with more severe stress into the severe anxiety cluster.

**Conclusions:**

The observed associations between stress and anxiety shed light on the interrelations between even very mild (subclinical) stress and anxiety symptoms and may point to the potential of mild stress to serve as a target for early interventions aiming to prevent anxiety morbidity.

## Background

Stress is a normal physiological and mental response to demanding circumstances [1]*.* It is expressed with both physical and emotional tension, triggered by multiple thoughts. Stress can even have positive sides, since it can exert catalytic effects on developing strategies to cope with challenges and stressors. Nonetheless, the higher the intensity and frequency of stress feelings, the more difficult it becomes for the individual to deal with activities of daily living. Intense and persistent stress affects professional, social, psychological functioning and undermines quality of life. Excessive or prolonged (but not severely) stressful situations and/or maladaptive person’s response capacities can induce emotional exhaustion and disengagement (burnout syndrome) or exacerbate mental disorders [2, 3]. Stress, particularly in early life, has been shown to exert a significant influence on risk of anxiety as well as further mental disorders (e.g. depression) [4, 5]. Interestingly, the new practical and easy-to-use Subclinical Stress Symptom Questionnaire- 25 (SSQ-25) enables a valid assessment and differentiation of even subclinical stress symptoms and their relevance in the study of psychopathological trajectories [6]. It is noteworthy, that acute stress- and post-traumatic stress disorder, forming the one extreme of the spectrum of stress responses, are not classified as anxiety disorders any longer according to the diagnostic guidelines of the Diagnostic and Statistical Manual of Mental Disorders-5 (DSM-5), despite the intuitive clinical relationship between stress and anxiety [7]. They are categorized into the new trauma- or stressor-related disorders, for the diagnosis of which the exposure to a traumatic event is required.

Anxiety disorders embody increasingly common mental illnesses [8–12]. Up to 33.7% of population worldwide are affected by an anxiety disorder during their lifetime. Anxiety is not less common among students [13], whilst the number of university employees seeking counselling and support from occupational health services has shot up [14]. In Greece, approximately 23% of a convenience sample of 901 adults was found to suffer from severe or extremely severe anxiety [15]. Anxious responses are expressed with psychological (e.g. feelings of fear, panic), behavioural (for instance, restlessness, irritability) and physiological (e.g. sweating, palpitations, tremor) symptoms. They embody a reaction to a stimulus related to real or perceived threat [7, 16]. The term perceived threat refers to stimuli, that are realistically not threating, harmful or uncontrollable and their nature, depth and duration do not justify anxious responses [17]. Anxiety disorders, such as panic disorder or generalized anxiety disorder, embody a crucial clinical issue, warranting therapeutic intervention, since in these cases anxiety feelings are in their quality and/or intensity so severe that they impair occupational and social functioning [18].

A considerable symptom overlap has been consistently observed between anxiety and depression. Depression is a major public health problem, since about 150 million people across the globe suffer from it at any moment [19]. In Greece, the prevalence of major depression increased from 3.3% to over 12% in the last decade possibly owing to the long lasting economic hardships [20]. Interestingly, 70% of individuals with mood disorders fulfil criteria for an anxiety disorder during their lifetime and up to 90% of patients with anxiety disorders fulfill criteria for concurrent mood disorder [21]. Anxiety and stress are common symptoms of a depressive episode [22, 23], while many antidepressant drugs have both antidepressant and anxiolytic effects) [24]. Approximately 30 years ago, a dimensional approach to depression and anxiety, the tripartite model, was proposed [25]. According to it, mood and anxiety disorders consist of a general distress factor, physiological hyperarousal (specific to anxiety) and anhedonia (specific to depression). According to a growing body of evidence, the pathogenesis of depression can be driven by internal biological processes linked to experiences of social-environmental stress [5]. In addition, anxiety and mood disorders have common genetic underpinnings and share abnormalities in task-related brain activity related to inhibitory control and salience processing such as right-sided hypoactivation in the inferior prefrontal cortex/insula, the inferior parietal lobule and the putamen [21, 24, 26].

The aim of the present study was to shed light on potential associations between stress, grasped by an instrument sensitive to detect even subclinical stress, i.e. SSQ-25, and anxiety symptoms not only in mentally healthy individuals of different age groups and occupations, but also in patients suffering from major depression. Its novelty hinges on the reliability of the SSQ-25 in detecting even subclinical stress and on the assessment of stress symptoms in a clinical sample with major depression with SSQ-25.

## Methods

### Participants

The current cross-sectional investigation included (1) a group of patients with major depression and a convenience sample of mental healthy individuals consisting of (2) undergraduate students, (3) postgraduate students and (4) employees of the University of Patras. The inclusion criteria for patients with major depression were willingness to participate in the study after detailed presentation of the study aims and procedures and a current episode of major depression. The exclusion criteria included the presence of a mental disorder other than major depression and/or incapacity to give written informed consent. The inclusion criteria for mentally healthy individuals were willingness to participate in the study and absence of a current episode of a mental disorder being treated with pharmaco- and/or psychotherapy. The exclusion criteria included being on psychotherapy and/or on medical treatment because of previous episodes of a mental disorder and/or incapacity to give written informed consent. The study was conducted between April 2018 and October 2019. Patients suffering from major depression fulfilled the DSM 5 criteria for major depression and were recruited at the afternoon outpatient clinic of the Department of Psychiatry of the Patras University General Hospital and at the Special Health Service Unit of the University of Patras, Greece. The diagnosis of major depression was established by an experienced psychiatrist who had examined the patient and had coordinated the diagnostic workup (e.g. brain imaging, neurocognitive assessment). The mentally healthy undergraduate students were students of the School of Humanities and Social Sciences of the University of Patras and were recruited at seminars and other didactic activities. Postgraduate students were recruited at the one-day health, safety and security seminars for first- year postgraduate students of the University of Patras. University employees were recruited through word-of-mouth. The study was approved by the Bioethics and Research Ethics Committee of the University of Patras. All procedures performed in the study were in accordance with the 1964 Helsinki declaration and its later amendments.

### Stress and anxiety assessment tools

#### SSQ-25

The SSQ-25 consists of 25-questions focusing on different aspects of stress symptoms within the last 4 weeks prior to its administration [6]. The questions are answered in a five-point Likert scale manner (from zero = not at all to five = very strong). The SSQ-25 captures psychological and physiological stress. The presence of psychological stress is assessed with 15 questions focusing on internal tension, nervousness, and further issues and concerns. Physiological stress is assessed with 10 questions concerning pain, weight changes, circulatory disturbances, insomnia. The constituent items of SSQ-25 have been chosen in such a way that stress symptoms laying below the threshold of clinical significance are also captured. The higher mean (standard deviation) total SSQ-25 score of the two via websites and companies’ mailing lists recruited cohorts on whom the initial report on SSQ-25 properties is based, was 37.1 (19.7). The instrument was translated into Greek by a bilingual member of our team. Thereafter, a bilingual expert not familiar with the original SSQ-25 made a back translation into German. The new version was very similar to the original one. The internal consistency (Cronbach’s α = 0.944) and the reliability (test-retest Intraclass Correlation Coefficient = 0.937) of the Greek version of the SSQ-25 were excellent.

#### State trait anxiety inventory Y (STAI Y)

The STAI Y is a short, valid and reliable, self - assessment scale that assesses both state- (s-) and trait (t-) anxiety, with 20 items, respectively. In the present study, STAI Y was employed for assessing t- anxiety, which refers to the stable individual proneness to anxiety feelings, which constitutes a personality trait [27]. Possible responses to each of the items grasping t- anxiety vary from almost never (one point) to sometimes (two points), often (three points) or almost always (four points). A rating of four indicates the presence of very high levels of anxiety for 10 items (# 2, 3, 4, 5, 8, 9, 11, 12, 14, 15, 17, 18 and 20). On the other hand, higher scores in the rest of the items indicate lower anxiety levels. The sum of these items is subtracted from the total sum. The score of the trait aspect of STAI Y varies between 20 and 80. Its application to the Greek population unveiled in a group of healthy individuals a mean (standard deviation) t-anxiety score of 27.88 (11.43). The reported data for the studied patient population suffering from anxiety were 43.50 (9.99) for t- anxiety [28, 29].

#### Beck anxiety inventory (BAI)

The BAI is one of the most widely used psychometric scales for assessing anxiety. It is a self-report questionnaire, consisting of 21 questions assessing how severe discomfort a person has experienced over the last week due to common anxiety symptoms, such as numbness and tingling, heat-induced sweating and fear of potential harm. Each item is rated on a scale of zero (not at all) to three (seriously). Higher overall scores indicate more severe anxiety symptoms: Scores between zero and seven points to minimal anxiety; eight to 15 points are compatible with mild anxiety; 16–25 points indicate moderate anxiety, while scores between 26 and 63 point to severe anxiety. Of note, BAI is designed to measure clinical anxiety [30, 31]. It has been translated into Greek and applied to Greek samples [32, 33]. In a non-clinical group, the mean (standard deviation) score was 11.02 (10.46), whilst in individuals beginning psychotherapy the mean (standard deviation) score was 19.7 (11.3) [32].

### Statistical analysis

Data were analysed using SPSS 23.0 for Windows. Descriptive statistics were generated for all variables. Statistics encompassed x^2^ test and ANOVA, as well as Scheffe post-hoc test for quantitative variables. The normal distribution of data was assessed using skewness and kurtosis. Linear regression analysis models were employed for studying the relationship between stress- and anxiety symptoms. A Step Cluster analysis using Log-Likelihood Distance measure and fixed number of two clusters was performed as an exploratory analysis that tries to identify homogeneous groups of cases within a dataset. Since it is exploratory, it makes no distinction between dependent and independent variables. Probability values of < 0.05 were considered to indicate statistical significance.

## Results

The demographic and clinical characteristics of the sample are presented in Table 1. In 12 STAI Y- and 10 BAI questionnaires, there were items which were not answered. In these cases, total scores were not calculated and not considered in the analyses. The groups did not differ with regard to sex distribution. Age differences attained statistical significance (ANOVA, *P* < 0.001). The scores of SSQ-25 varied significantly among the four groups (ANOVA, *P* < 0.001). Stress symptoms did not differ between undergraduate students and patients with major depression, whilst stress levels of both groups were significantly higher compared to postgraduate students. The lowest stress levels were detected in the latter group. Anxiety symptoms of undergraduate students and patients with depression were comparable. Their symptoms were more severe compared to the groups of postgraduate students and employees. Of note, the scores of the trait aspect of STAI Y did not differ across the groups (*P* = 0.37).

**Table 1:** Demographic and clinical data of the study sample

	Undergraduate Students	Postgraduate Students	Employees	Patients with depression
N	233	71	108	77
Age (in years)^a^	21,65 (2,46) ^**##††^	26,41 (5,01) ^##††^	40,66 (9,96)^††^	31,42 (13,58)
Sex: female (%)	158 (49,4)	43 (13,4)	62 (19,4)	57 (17,8)
SSQ-25^a^	63.01 (16.65)^**#^	47.92 (17.35)^#†^	56.48 (21.35)	63.10 (15.56)
BAI^a^	*(N = 232),* 38.97 (9.79)^**##^	32.41 (8.78)^†^	*(N = 99),* 33.91 (11.16)^†^	38.43 (9.66)
STAI Y trait aspect^a^	*(N = 231),* 46.71 (6.16)	47.87 (6.19)	(*N = 99),* 47.67 (5.23)	46.82 (6.56)

^a^Data presented as mean (standard deviation); SSQ-25: Subclinical stress symptom questionnaire 25; BAI: Beck anxiety inventory; STAI Y trait aspect: State trait anxiety inventory Y trait subscale

Posthoc multiple comparisons of quantitative data were conducted with Scheffe test

**statistically significant differences in mean value in comparison to postgraduate students (*P* ≤ 0.001)

#statistically significant differences in mean value in comparison to employees (*P* < 0.05)

##statistically significant differences in mean value in comparison to employees (*P* ≤ 0.001)

†statistically significant differences in mean value in comparison to patients with depression (*P* < 0.05)

††statistically significant differences in mean value in comparison to patients with depression (*P* ≤ 0.001)

As to the relationship between stress and anxiety symptoms, the SSQ-25 scores were found to be associated with the BAI questionnaire. The regression model, using BAI score as the dependent variable (adjusted R^2^ = 0.55, F = 193.06, *P* < 0.001), revealed a significant impact of SSQ-25 score on anxiety levels (standardised partial regression coefficient of SSQ-25 = 0.75, *P* < 0.001), whereas age (standardised partial regression coefficient of age = 0.01, *P* = 0.70) and group identity (standardised partial regression coefficient of group identity = 0.03, *P* = 0.43) did not exert any significant influence. Furthermore, in order to explore possible influence of interactions between group identity and SSQ-25 on anxiety symptoms, a group identity x SSQ-25 interaction parameter was entered as an additional explanatory variable into the aforementioned linear regression model. However, the total variance explained was not increased (adjusted R^2^ = 0.55, F = 144.49, *P* < 0.001) and hence, the association of the interaction parameter with anxiety levels was not significant (standardised partial regression coefficient of group identity = 0.01, *P* = 0.95).

Based on a two- step cluster analysis using Log-Likelihood Distance measure and fixed number of two clusters, the entire group was divided into two clusters differing in the severity of stress symptoms. The mean (standard deviation) SSQ-25 score of the cluster with milder (subclinical) stress symptoms which included 77 participants was 34.29 (7.84), whilst that of the second cluster was almost double, i.e. 63.77 (15.77). The mean (standard deviation) ΒΑΙ score of the cluster with milder stress symptoms was 22.92 (1.33) indicating moderate anxiety, whilst that of the second cluster was almost double, i.e. 39.61 (8.85) pointing to severe anxiety. The Two Step Cluster analysis achieved to classify 76/77 participants with milder stress symptoms into the cluster with moderate anxiety and 400/400 participants of the second stress cluster into the severe anxiety cluster.

## Discussion

The main finding to emerge is the positive association between stress levels and anxiety independently of the severity of stress symptoms. The new instrument SSQ-25 is a valuable and reliable tool for detecting subclinical stress. Its subclinical property has recently been confirmed by means of item information functions, scatter plots, residuals and Koenker-Basset test [6]. Interestingly, the study participants who were classified into the cluster with the less severe stress in the Two Step Cluster analysis, manifested stress symptoms comparable in terms of severity with that of previously described cohorts with subclinical stress [6]. The positive correlation between BAI- and SSQ-25 scores was statistically significant independently of group identity. Of note, stress correlated significantly with anxiety even in the group of postgraduate students, being the study group which was less inflicted by stress (data not shown). Even though grasping stress severity seems to indicate anxiety levels too, the observations of the present cross-sectional study do not establish any straightforward causal effect of stress on the development of anxiety symptoms, especially in the light of the catalytic effects of anxiety personality trait on expression of higher levels of perceived stress for exposure to the same stressor and on development of stress-induced psychopathological changes (e.g. depression, sleep fragmentation, anxiety) [17, 27]. Α causal relationship can only be established through longitudinal experimental studies aiming to elucidate the impact of stress reduction on anxiety levels.

The observed positive association between stress and anxiety symptoms is underpinned by neurobiological linkages. Over the past two decades functional imaging studies have unveiled multiple brain regions including the hypothalamus, amygdala, prefrontal cortex and nuclei of the brainstem which are active during both stress and anxiety responses in healthy individuals [7, 34]. The locus coeruleus noradrenergic system pertains to both physical and emotional responses to stress, since noradrenaline release leads to a state anxiety response. This brain region is linked to neurons in the basolateral amygdala that project to the ventral hippocampus and to the medial section of the central amygdala, two brain regions crucially implicated in anxiety-related behaviours. In addition, accumulating evidence points to the role of hypothalamic corticotropin neurons in the modulation of behaviour under stressful circumstances. Being pivotal players in stress and anxiety linkages, these neurons control the way an individual deals with stress exposure, are involved in the regulation of anxiety-like behaviours as also in the social transmission of stress [7, 34].

Not all groups of mentally healthy individuals significantly differed in anxiety and stress levels from patients suffering from major depression. As expected, stress and anxiety levels of patients with major depression were far beyond subclinical levels with BAI scores pointing to severe anxiety [21]. In comparison to them, stress levels were significantly lower only in the postgraduate student group. Quite unexpectedly, undergraduate students and university employees were found to exhibit comparable levels of stress and anxiety with that of patients with depression. This observation cannot be attributed either to bias stemming from differences between the groups in anxiety trait, since the trait aspect of STAI Y did not differ across the groups, or to ceiling effects of SSQ-25 taking into account its focus on subclinical stress. Total SSQ-25 scores in patients with major depression were far below the highest possible score. In line with our observation, a rapid increase in yearly visits to university counseling centers has been recently reported [35, 36], whilst prevalence rates of anxiety disorders in students seem to be approximately 15% [37, 38] or even higher [13] and medical and nursing studies pertain to significant psychological distress [39, 40]. Anxiety disorders in students are associated with a precarious job or unemployment of the father of the student, a constellation not so rare in Greece, a country which has been scourged by a deep socioeconomic crisis for several years [37, 41]. The financial crisis and the impact of economic stress (financial deterioration and insecurity) could also to same extent explain the relatively high stress levels observed in the group of university employees [15, 42]. Despite being significantly lower compared to undergraduate students’ stress, their stress levels did not differ from that of patients with depression. In line with our findings, previous reports point to high stress and anxiety levels in university staff, too [14]. Of note, being followed-up by a psychiatrist and/or psychologist, which was an exclusion criterion for mentally healthy individuals in the present study, has been recently found to be associated with older age in under- and postgraduate medical students [43]. Thus, it can be speculated that postgraduate students with relatively severe stress and anxiety symptoms, who had not suffered from major depression, had sought medical/psychological advice and treatment and consequently were not eligible for enrollment in the present study.

Except for the difference in stress between university employees and postgraduate students, all further detected significant differences in stress levels were coupled with significant differences in anxiety levels. Contrary to the detected significantly higher levels of stress in university employees compared to postgraduate students, anxiety levels did not differ across these two groups. This decoupling warrants further investigation. It may be attributed to differences between the groups in the nature, duration and/or the depth of the stressors to which they are exposed, as well as in coping strategies [44].

Several limitations should be taken into account when interpreting the findings of the present study. First, psychological and physiological stress responses were not considered separately. Although they are characterized by complex feedback mechanisms and interrelations, they may develop quite independently, as clearly mirrored in the largely weak correlation between psychological and physiological stress responses [45]. The type of response is shaped by a complex interplay between the type of stressor, social evaluation, which is contingent on individual subjective appraisal and personal experiences, and demographic, biological and psychological characteristics such as gender, menstrual cycle phase, use of oral contraceptives, age, body weight, personality traits [46]. Since the nature, depth and duration of the stressors being in play in each participant were not tapped in the present study, and no biological markers of physiological stress such as salivary cortisol or plasma adrenaline or noradrenaline levels were determined, physiological and psychological stress were not considered separately here, in order to minimize potential sources of bias. Second, despite the moderating effects of negative metacognitive beliefs on the interrelation between perceived stress and anxiety [47], metacognitive measures (e.g. meta-cognitive questionnaire-30) were not employed and hence, negative metacognitive beliefs were not included as independent variable in the analyses. Third, personal and family history of traumatic and stressful life events such as childhood abuse or neglect were not examined. Fourth, unfortunately depressive symptoms were not captured with a depression scale (e.g. Hamilton Depression Rating Scale or the Beck Depression Inventory) either in patients who suffered from major depression or in mentally healthy individuals. Despite the high diagnostic validity of modern diagnostic criteria for major depression [23], the presence of subclinical or very mild depressive symptoms, not justifying the diagnosis of a clinical depressive or anxiety disorder, cannot be precluded. Recently, high proneness of medical students with high levels of trait anxiety to clinical depression and particularly to persistence of depressive symptoms was reported [48]. Even though, the levels of trait anxiety in the groups of the present study were relatively high [28], it is underscored that the presence of a clinical depressive or other mental disorder was an exclusion criterion for recruiting mentally healthy individuals. Fifth, in a number of BAI- and STAI Y questionnaires not all items were answered, so that the respective total scores were neither calculated nor considered in the analyses. Moreover, the focus of our study exclusively on students, university employees and patients with major depression, constrains the generalizability of our observations. In addition, due to the cross-sectional nature of the study, no definite conclusions can be drawn with regard to the potential of stress reduction as an anxiety prevention strategy. Future studies with a longitudinal, experimental design and larger sample size, as also studies based on brain imaging could provide the necessary evidence for the potential of mild stress to serve as a target for primary interventions aiming to prevent anxiety morbidity in the general population.

## Conclusions

To conclude, the line of research on the relationship between stress and anxiety is here extended in the way that the findings of the present study indicate an association between anxiety and even subclinical stress symptoms, which were assessed with the new and valid instrument SSQ-25. Though the cross-sectional design of the study, precludes conclusions regarding the direction of this association, our observations may point to the usefulness of interventions aiming to ameliorate even subclinical stress symptoms among individuals who do not suffer from a mental disorder but face difficulties to deal with stressful situations.

## Data Availability

The datasets used and analyzed during the current study are available from the corresponding author on reasonable request.

## Abbreviations

SSQ-25: Subclinical stress symptom questionnaire 25
BAI: Beck anxiety inventory
STAI Y: State trait anxiety inventory
s- anxiety: State anxiety
t- anxiety: Trait anxiety
DSM-5: The Diagnostic and Statistical Manual of Mental Disorders, Fifth Edition
ANOVA: Analysis of variance
